# An XPS Study of Electrolytes for Li-Ion Batteries in Full Cell LNMO vs Si/Graphite

**DOI:** 10.1021/acsami.4c01891

**Published:** 2024-06-21

**Authors:** Raheleh Azmi, Fredrik Lindgren, Killian Stokes-Rodriguez, Mihaela Buga, Cosmin Ungureanu, Tom Gouveia, Ida Christensen, Shubhadeep Pal, Alexandru Vlad, Alix Ladam, Kristina Edström, Maria Hahlin

**Affiliations:** † Department of Chemistry − Ångström Laboratory, Structural Chemistry, Uppsala University, Box 538, Uppsala 751 21, Sweden; ‡ Department of Sustainable Energy Technology, SINTEF Industry, Trondheim 7491, Norway; § ROM-EST Laboratory, ICSI Energy Department, National Research and Development Institute for Cryogenic and Isotopic Technologies − ICSI, 4 Uzinei, Ramnicu Valcea 240050, Romania; ∥ Faculty of Energy Engineering, National University of Science and Technology POLITEHNICA Bucharest, 313 Splaiul Independentei, Bucharest 060042, Romania; ⊥ Research and Innovation Department, Solvionic, Toulouse 31100, France; # Vianode, Kristiansand 4621, Norway; ∇ Institute of Condensed Matter and Nanosciences, Université Catholique de Louvain, Louvain-la-Neuve B-1348, Belgium; ▼ Department of Physics and Astronomy, Division of X-ray Photon Science, Uppsala University, Box 516, S-751 20 Uppsala, Sweden

**Keywords:** LNMO-Si/graphite battery, solid electrolyte interface, SEI, cathode electrolyte interface, CEI, surface analysis, ionic liquid electrolyte

## Abstract

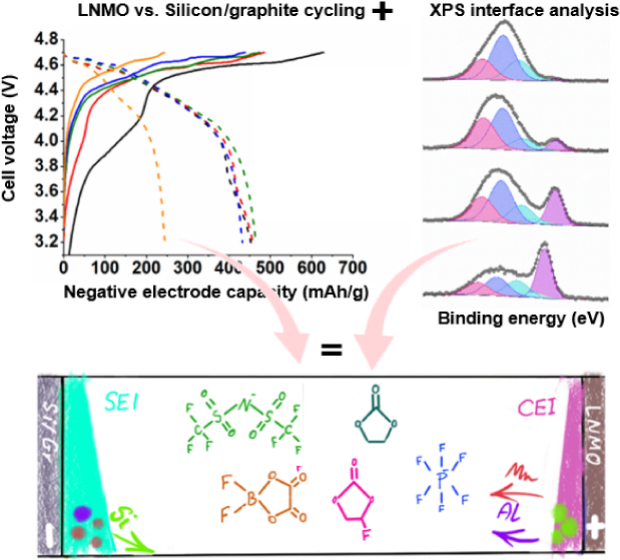

Two different types of electrolytes (co-solvent and multi-salt) are tested for use in high voltage LiNi_0.5_Mn_1.5_O_4_||Si/graphite full cells and compared against a carbonate-based standard LiPF_6_ containing electrolyte (baseline). Ex situ postmortem XPS analysis on both anodes and cathodes over the life span of the cells reveals a continuously growing SEI and CEI for the baseline electrolyte. The cells cycled in the co-solvent electrolyte exhibited a relatively thick and long-term stable CEI (on LNMO), while a slowly growing SEI was determined to form on the Si/graphite. The multi-salt electrolyte offers more inorganic-rich SEI/CEI while also forming the thinnest SEI/CEI observed in this study. Cross-talk is identified in the baseline electrolyte cell, where Si is detected on the cathode, and Mn is detected on the anode. Both the multi-salt and co-solvent electrolytes are observed to substantially reduce this cross-talk, where the co-solvent is found to be the most effective. In addition, Al corrosion is detected for the multi-salt electrolyte mainly at its end-of-life stage, where Al can be found on both the anode and cathode. Although the co-solvent electrolyte offers superior interface properties in terms of the limitation of cross-talk, the multi-salt electrolyte offers the best overall performance, suggesting that interface thickness plays a superior role compared to cross-talk. Together with their electrochemical cycling performance, the results suggest that multi-salt electrolyte provides a better long-term passivation of the electrodes for high-voltage cells.

## 1 Introduction

The higher energy density required for the growing electro-mobility and renewable energy storage markets can potentially be delivered by utilizing silicon-containing anodes and insertion-type cathodes.^1^ The high specific capacity of Si (3579 mA h g_Si_^–1^ upon Li_3.75_Si formation) as well as its low cost and low lithiation potential (∼0.4 V vs Li/Li^+^) acts as a good driving force for its usage to this purpose.^2^ Also, among several Li-containing insertion-type cathodes such as LiFePO_4_ (∼3.4 V vs Li/Li^+^), and LiCoO_2_ or LiNi_0.33_Mn_0.33_Co_0.33_O_2_ (∼3.7 V vs Li/Li^+^), the LiNi_0.5_Mn_1.5_O_4_ (∼4.8 V vs Li/Li^+^) spinel cathodes, with their high operating potential and structural stability, make a good choice for successful development of future high energy density batteries.^1^ However, there are several challenges in realizing long-life and safe batteries for the chemistry mentioned above for commercial uses.

Some of these challenges are related to finding a suitable electrolyte system that has a stable electrode/electrolyte interface on both high-capacity negative and high-voltage positive electrodes. The stability of an electrolyte in a battery is determined by the relationship between its reduction and oxidation potentials relative to the Fermi energy levels of the positive and negative electrodes. For a stable electrolyte, the electrochemical potentials of the electrodes must fall within the electrolyte’s stability window. However, this is rarely the case, and the formation of the passivating solid electrolyte interface (SEI) layer is a well-studied mechanism that enables the negative electrode to operate outside the stability of the electrolyte.^3^ Maintaining the passivating properties of SEI is particularly challenging for the alloying-type high-capacity negative electrodes such as Si. That is because the enormous volume expansion of Si-containing negative electrodes upon lithiation induces mechanical degradation of the SEI and, with successive cycling, continuous consumption and drying of the commonly used organic carbonate-based electrolytes.^1,2^ Also, on the positive electrode side, the increasing demand for higher voltages approaches the upper limits of the stability window of most conventional electrolytes. Here, the cathode electrolyte interface (CEI) will form as a consequence of electrolyte and active material decomposition,^4^ which makes these types of electrolytes^1,2^ a less appropriate choice for high-energy density batteries.^5^

A scalable mitigation strategy to deal with volume expansion and unstable SEI/CEI layers (thus improving Coulombic efficiency (CE)) is the development of electrolyte additives that possess multiple functionalities.^1^ One functionality of the additives is to act as SEI/CEI builders and to catch protonic species.^1^ Fluoroethylene carbonate (FEC) is a common sacrificial SEI builder additive. It has been reported that it functions by being defluorinated and decarboxylated on anodes before the decomposition of common organic carbonate solvents and forming a highly elastomeric polymer network of likely polyvinyl carbonate, resulting in a thinner and more flexible SEI that better buffers the volume expansion/contraction of the silicon-containing anodes.^6,7^ However, an important shortcoming of FEC is its Lewis-acid-induced defluorination, resulting in the formation of HF and HPO_2_F_2_ that in turn accelerate the degradation of lithium hexafluorophosphate (LiPF_6_)-based electrolytes (besides HF being highly toxic and corrosive to the environment).^1,8^ Moreover, the oxidative decomposition of FEC is found to harm the long-term stability of battery systems using high-voltage LiNi_0.5_Mn_1.5_O_4_ cathodes.^9^ Despite this, small amounts of FEC were incorporated into the electrolytes in this work because of its effective SEI-forming attributes for Si-based anodes, as no better alternatives were found to mitigate its drawbacks.

As of now, additives cannot combat the decomposition of the LiPF_6_ salt. Besides its advantages, this commonly used salt is the origin of many degradation phenomena in the Li-ion cells. The hydrolytic instability of LiPF_6_ produces HF. These reactions summarize the HF formation mechanism: LiPF_6_ ↔ LiF + PF_5_; PF_5_ + H_2_O ↔ 2HF + POF_3_ and then POF_3_ + H_2_O ↔ HF + HPO_2_F_2_.^10,11^ Among other sources of moisture, for Si/graphite (Si/g) cells, lithium polyacrylate (LiPAA), and other aqueously processed polycarboxylate binders can introduce water to the cells.^12,13^

The decomposition of LiPF_6_ in turn is the trigger of many other fatal mechanisms such as Si and transition metal etching/dissolution resulting in cell failure.^10,14^ Mn dissolution occurs more often than other transition metals, and its accumulation on the anode triggers irreversible side reactions that lead to continuous electrolyte reduction, SEI impedance growth, and the loss of cyclable lithium.^15^ Besides the etching of high valence Mn cations by HF acid, the Jahn–Teller effect also causes a disproportionation reaction of Mn^3+^ (2Mn^3+^ → Mn^4+^ + Mn^2+^).^16^

Another important class of SEI builder additives is ionic liquids that can stabilize SEI/CEI via cation and/or anion functionalities. Lithium ionic liquid salts such as lithium bis(oxalato)borate (LiBOB), lithium difluoro(oxalato)borate (LiDFOB), and lithium fluoromalonato(difluoro)borate (LiFMDFB) can also act as HF and HF_2_O_2_ trappers, in addition to SEI/CEI building, making the battery relatively environmentally friendly. On the other hand, lithium bis(fluorosulfonyl)imide (LiFSI), lithium 2-trifluoromethyl-4,5-dicyanoimidazole (LiTDI), and their modifications can replace LiPF_6_. Such salt anions can effectively build LiF and Li_3_N-rich SEI with their electrochemically active covalent bonds (e.g., S–F, S–N), also contributing to relatively green and safer electrolytes.^1,17^ LiF is known for its high stability and excellent electrical insulating properties; therefore, it can improve interface stability; however, excessive LiF can instead hamper the Li^+^ transfer.^18^ Therefore, there is always a need for careful optimization of the electrolyte components. This is particularly important knowing that both LiFSI and lithium bis(trifluoromethanesulfonyl)imide (LiTFSI), in combination with nonfluorinated carbonates, can cause cathode aluminum current collector corrosion.^19^ It is hypothesized that the formation of a protective aluminum (oxy-)fluoride layer on the aluminum current collector surface in electrolytes containing F^–^ ions can stop this corrosion.^20^

In this paper, with all the above-mentioned challenges in mind, we cycle lithium nickel manganese oxide (LNMO) positive electrodes vs Si/g negative electrodes using selected electrolytes and investigate changes in the surface and interphase chemistry using X-ray photoelectron spectroscopy (XPS) during the battery life span. More specifically, we track the atomic-level effect of some of the mitigation strategies that should improve cycle life and capacity retention of such cells by adding an ionic liquid co-solvent or utilizing a multiple Li-salt system. We compare the surface layer composition and electrolyte degradation products obtained from XPS analysis on these designed electrolytes to a traditional single salt in an organic solvent electrolyte system.

## 2 Experimental Details

### 2.1 Electrode Preparation, Fabrication, and Electrochemical Measurement

Lithium nickel manganese oxide (LiNi_0.5_Mn_1.5_O_4_, LNMO) powder (Johnson Matthey), used as active material, was mixed with the PVdF binder (Solvay Solef 5130) (5% solution in NMP, *N*-methyl-2-pyrrolidone, from Sigma-Aldrich) and conductive additive C65 (Imerys), according to the following formulation: 90 wt % LNMO, 5 wt % PVdF, and 5 wt % C65, in a double-walled water-cooled stainless-steel container using a Vacuum planetary mixer TOB-XFZH01. One-side-coated cathode was manufactured using the roll-to-roll coating machine (Thank Metal) available at the ICSI R&D pilot line. The slurry was deposited on a 20 μm battery grade aluminum current collector (Xiamen TOB New Energy Technology Co., Ltd). Then, the electrode passed through an oven to remove any solvent trace and was finally calendered using double-roll calender equipment (Thank Metal). 13 mm diameter circular electrodes were punched using a high precision cutting plier (EL-Cut from El-Cell) and vacuum-dried overnight at 110 °C. The average mass of active material, after drying, was approximately 11 mg cm^–2^. Electrode disks were transferred into an argon-filled glovebox for pouch cell design assembly.

Negative electrodes were prepared using 92% Si/graphite (Si/g) composite (graphite:silicon, 85:15) provided by Vianode, 3% Na-CMC and 3% SBR (in a 15 wt % solution), and 2% carbon black C45. This was mixed in a centrifugal mixer together with water and cast onto a Cu foil. The resulting electrodes had a mass loading of approximately 1.3–1.4 mg cm^–2^. Electrodes were dried at 120 °C inside an argon-filled glovebox.

Cells were assembled in an argon-filled glovebox using a Celgard 2325 separator, and one of the three electrolytes is described in Table 1. The cells were sealed in aluminum pouches with a plastic lining under a vacuum.

**Table 1:** Electrolyte Composition and Nomenclature Used in This Work

composition	abbreviation
0.5 M LiTFSI, 0.5 M LiDFOB, 0.2 M LiPF_6_ in EC:EMC (3:7) + 10% FEC	multi-salt
1.2 M LiPF_6_ in (60 wt %EC:EMC (3:7) + 40 wt % PYR_13_FSI)+ 10% FEC	co-solvent
1.2 M LiPF_6_ in EC:EMC (3:7) + 10% FEC	baseline

Electrochemical cycling was carried out on a Land potentiostat with a voltage window between 4.7 and 3.2 V. First, two formation cycles used a current corresponding to C/20 (based on the negative electrode capacity), and the following two cycles used a current of C/10. Thereafter, 20 cycles of C/5 were employed, followed by another two cycles at C/20. The last two steps were repeated to reach the desired number of cycles.

### 2.2 X-Ray Photoelectron Spectroscopy (XPS)

The XPS measurements are performed on a Kratos AXIS Supra+ X-ray photoelectron spectrometer. Spectra were acquired using a monochromatic Al Kα (1487 eV) source operating at 144 W power (12 mA × 12 kV) and pass energies of 20 eV for high-resolution spectra and 160 eV for survey spectra. Spectra were acquired in hybrid spectroscopy mode over an area of approximately 700 × 300 μm. Charge compensation was achieved by using the Kratos electron charge compensation system. Measurements were performed directly on the electrodes.

The cycling of the cells was stopped after the first formation cycle, after 10 or 100 cycles, or at the end-of-life (EoL). Cells were then disassembled in an argon-filled glovebox. The electrodes were rinsed in 100 μL of DMC by loosely holding the electrode and gently dispensing the DMC from a pipet onto it. This allowed any excess salt to dissolve in the DMC. Subsequently, the rinsed electrodes were mounted onto double-sided Cu tape on an XPS sample holder. The cycled samples were then transferred under an inert atmosphere to the XPS analysis chamber. During XPS measurements, all samples were insulated from the spectrometer ground and charge compensated by using the flood gun of the spectrometer.

Data acquisition was carried out using the ESCApe software, and processing was performed with CasaXPS software (v 2.3.17), utilizing a Shirley background. Peak fits were achieved using the Gaussian–Lorentzian summation function (SGL), with a 90% Gaussian contribution except for graphitic sp^2^ hybridization, where the Gaussian contribution was fixed to 70%. The binding energies for the negative electrode are reported in reference to the C 1s peak of graphitic sp^2^ hybridization at 284.4 eV for pristine samples and the C 1s peak of hydrocarbons at 285.0 eV for cycled electrodes. The spectra of positive electrodes are referenced to the C 1s peak of PVdF at 290.8 eV (C–F). Later, with the advancement of the cycling, this peak may have some contribution from other CO_3_ and C–F-containing species; however, the alignment of other spectral lines confirms this to be a reasonable calibration point. Peak assignments in the deconvolution of the core level spectra can be seen in Table 2. The full width at half-maximum (fwhm) of the deconvoluted peaks does not exceed 1.8 eV and varies mainly by ±0.2 eV within a specific series of the samples. In some spectra with lower signal-to-noise ratios, a slightly higher fwhm is allowed, and these fittings are essentially used to estimate the peak areas for the calculation of elemental ratios. In the case of the other broader peaks such as Si 2p and N 1s, it is known that they can be a superposition of different decomposition components, and such peaks are indicated with dotted shades in the spectra.

**Table 2:** XPS Peak Binding Energy and Assignments

spectra	binding energy	assignment	references
C 1s	284.5	C–C (sp^2^) (cathode)	(21−23)
	285–286	C–H peak of PVdF, other hydrocarbon C–H	(21−23)
	286.5	C–O, C–N	(21−23)
	288	C=O	(21−23)
	290–293	CO_3_,^24^ C–F peak of PVdF, CF_3_ peak of LiTFSI	(21−23)
F 1s	684.5–685.5	LiF	(21−23)
	687–688	PVdF/LiPF_6_/Li_*x*_PF_*y*_O_*z*_/S–F	(21−23,25,26)
	689–690	CF_*x*_	(21−23)
O 1s	529, 530	Li–O LNMO	(24,27),^28^
	532–534	C–O/C=O/S=OP–O, B–Osalts and their decomposition products	(21−23,25,26,29−31),^32^
N 1s	400	LiTFSI/LiFSI	(32)
	402.8	PYR_13_	(33)
S 2p	164–167	decomposition products of LiTFSI/LiFSI	(34−36)
	170–171	LiTFSI/LiFSI	(32)
P 2p	134.5	oxidized species (Li_*x*_PF_*y*_O_*z*_)	(37,38)
	136.5	LiPF_6_	(39,40)
Si 2p	98.5	elemental Si	(21−23)
	101.5–102.5	SiO_*x*_	(21−23)
	104.5	Li_2_SiF_6_ species or Li_2_SiO_4_ species	(41−46)
Li 1s	50	overlapping Mn 3p peak	(47,48)
	54.5	Li in LNMO	(27,28)
	55.5–57.5	Li in SEI/CEI	(24)

## 3 Results

### 3.1 Electrochemical Cycling

**Figure 1:**
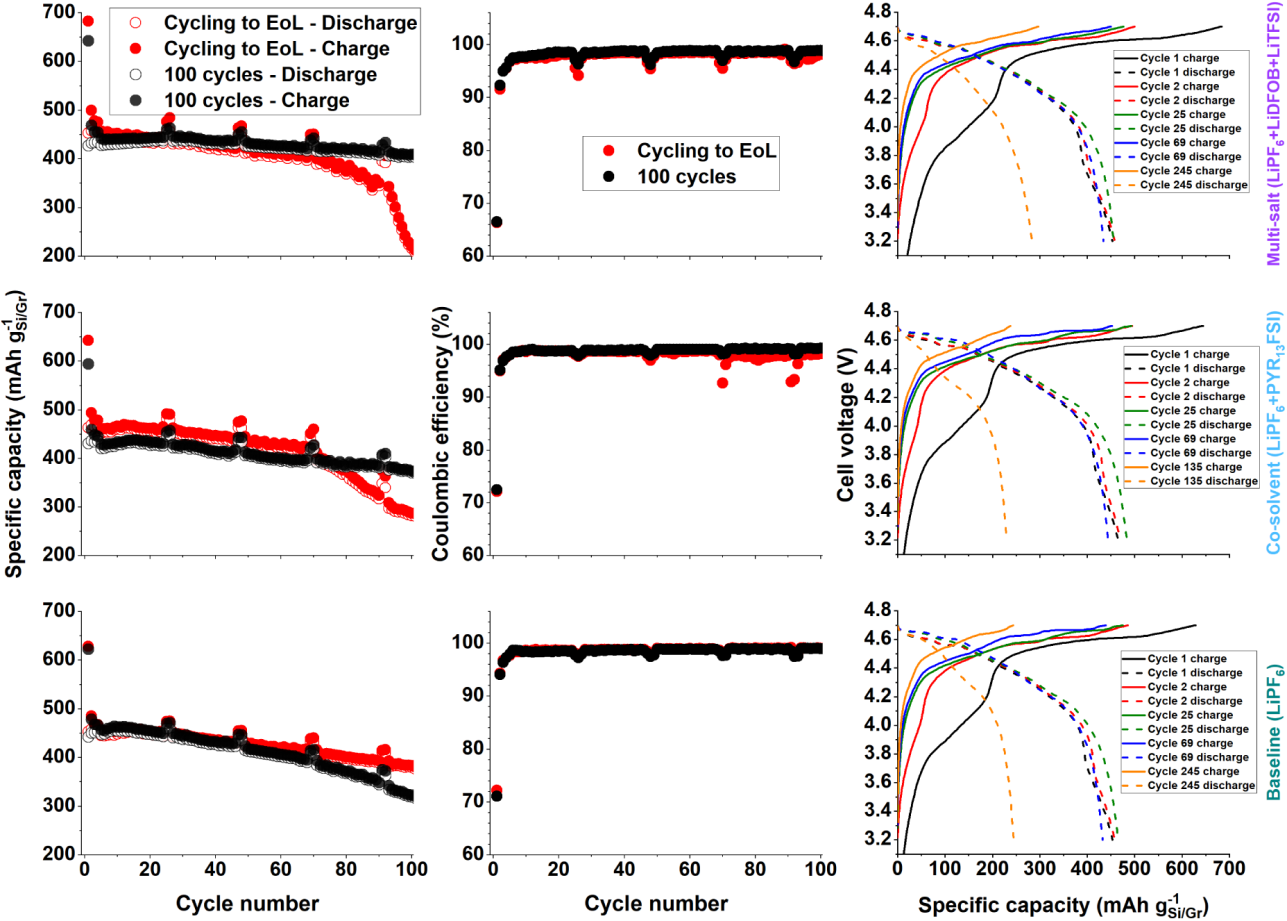
Specific capacity and Coulombic efficiency (based on the mass of the negative electrode) as a function of cycle number. Last column: voltage profiles of selected cycles (cell voltage over specific capacity).

The specific capacity (based on the Si/g electrode) and Coulombic efficiency of Si/g||LNMO full-cells using the different electrolyte compositions and cycled either for 100 cycles or to end-of-life (EoL), respectively, are shown in Figure 1 (right and center). The charge capacity in the first cycle is in the range of 595–683 mA h g^–1^ of Si/g of active material for all cells, with two parallel cells for each test investigated in this study. The displayed data represents the cell with the highest specific capacity for each electrolyte, in this work. The capacity retention varies to some extent between different electrolytes; however, a significant capacity fade is generally observed over the first hundred cycles for all cells, and the data also show a breakpoint in the cycling stability for end-of-life cells using the multi-salt electrolyte and the co-solvent electrolyte.

Even if the cycling performance varies between the long cycled cells (100 cycle and EoL samples), the Coulombic efficiency presents more similar values for both cells in the early cycles, where the two cells using multi-salt electrolyte have a first cycle CE of 66.5 and 66.3%. The equivalent values for the ionic liquid co-solvent are 72.2 and 72.5%, and for the baseline electrolyte, it is 71.1 and 72.2%, respectively. These values increase and reach a fairly stable value of 98% after 10 cycles and continue to increase over the first hundred cycles to approach 99% for all electrolytes.

The plateau around 3.8–4.2 V in the voltage profiles, see Figure 1 (right), stems from the LNMO material, and this plateau is shortened upon subsequent cycles. This is most likely due to irreversible SEI formation on the negative electrode, which in turn causes the amount of Li available for reinsertion into the LNMO to decrease.^12^ The LNMO material will have to utilize Li at higher voltages in subsequent cycles, and the voltage window of the LNMO will have to increase. This effect is most prominent in the early cycles where SEI formation is most active; however, its manifestation persists throughout the entire cycle life, by an observable increase in lithiation potential in the overall voltage profile.

The voltage profiles in Figure 1 show that the charge cutoff in this system occurs at a plateau, i.e., both the negative and the positive electrodes are in a relatively flat voltage region when the upper cutoff is reached. This means that any small changes in the voltage profile or the polarization of the system could have a major impact on the obtained charge capacity. This is because the upper cutoff voltage is reached before the full delithiation of the cathode. An increase in polarization could be the reason for the relatively unstable and rapidly reducing specific capacity observed for certain cells, as noted in Figure 1. Additionally, toward cell EoL, when the Li-reserves in the LNMO are running low (due to lithium losses in the continuous SEI formation), this will mean that LNMO will continuously have to increase its delithiation plateau to higher values, and it is likely that the potentials of the LNMO might exceed 4.9 V vs Li^+^/Li (which is usually considered the upper voltage limit for LNMO). This means that the LNMO electrode material and the electrolytes will have to deal with higher potentials, which is expected to increase the driving forces for electrolyte decomposition at the cathode, leading to even further accelerated cell aging.

During the initial charge cycles, the selected voltage profiles in Figure 1 also show a significant shortening of the plateau at 3.8–4.2 V. It is generally challenging to reveal the origin of changes in the full cell cycling curve since they contain the sum of the voltage profile from the negative and the positive electrode. Nonetheless, a significant change to all systems is observed in the first cycles, where gradually the plateau between 3.8 and 4.2 V disappears.

### 3.2 Surface Analysis with XPS

To further understand the capacity fade mechanisms of the cells, we performed XPS measurements on pristine and cycled positive and negative electrodes, as well as on the electrodes at the EoL stage. Particular focus is placed on the evolution of the SEI/CEI and metal dissolution from both the anode and cathode through cross-talk.

#### 3.2.1 Positive Electrode Observations

**Figure 2:**
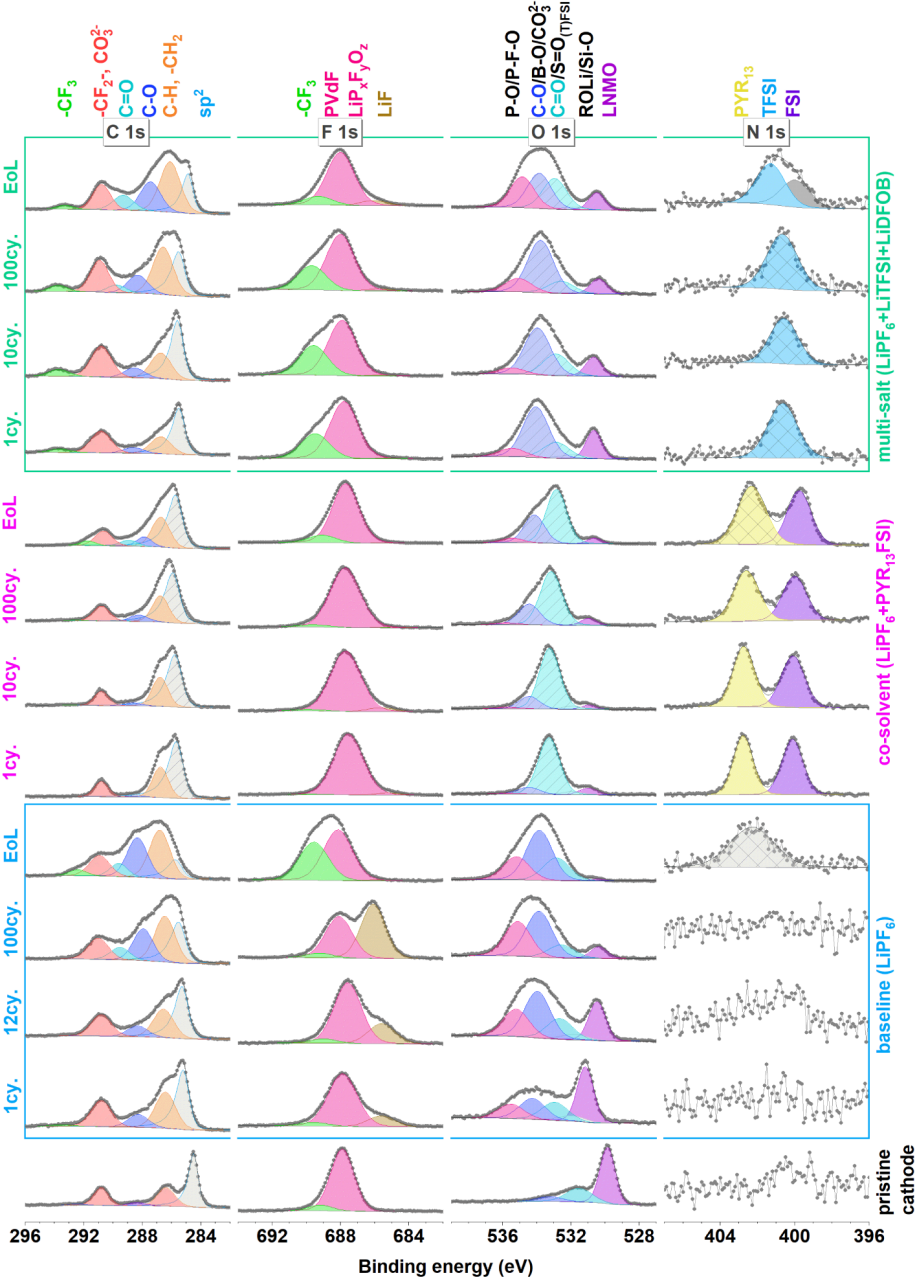
C 1s, F 1s, O 1s, and N 1s spectra of all positive electrodes.

**Figure 3:**
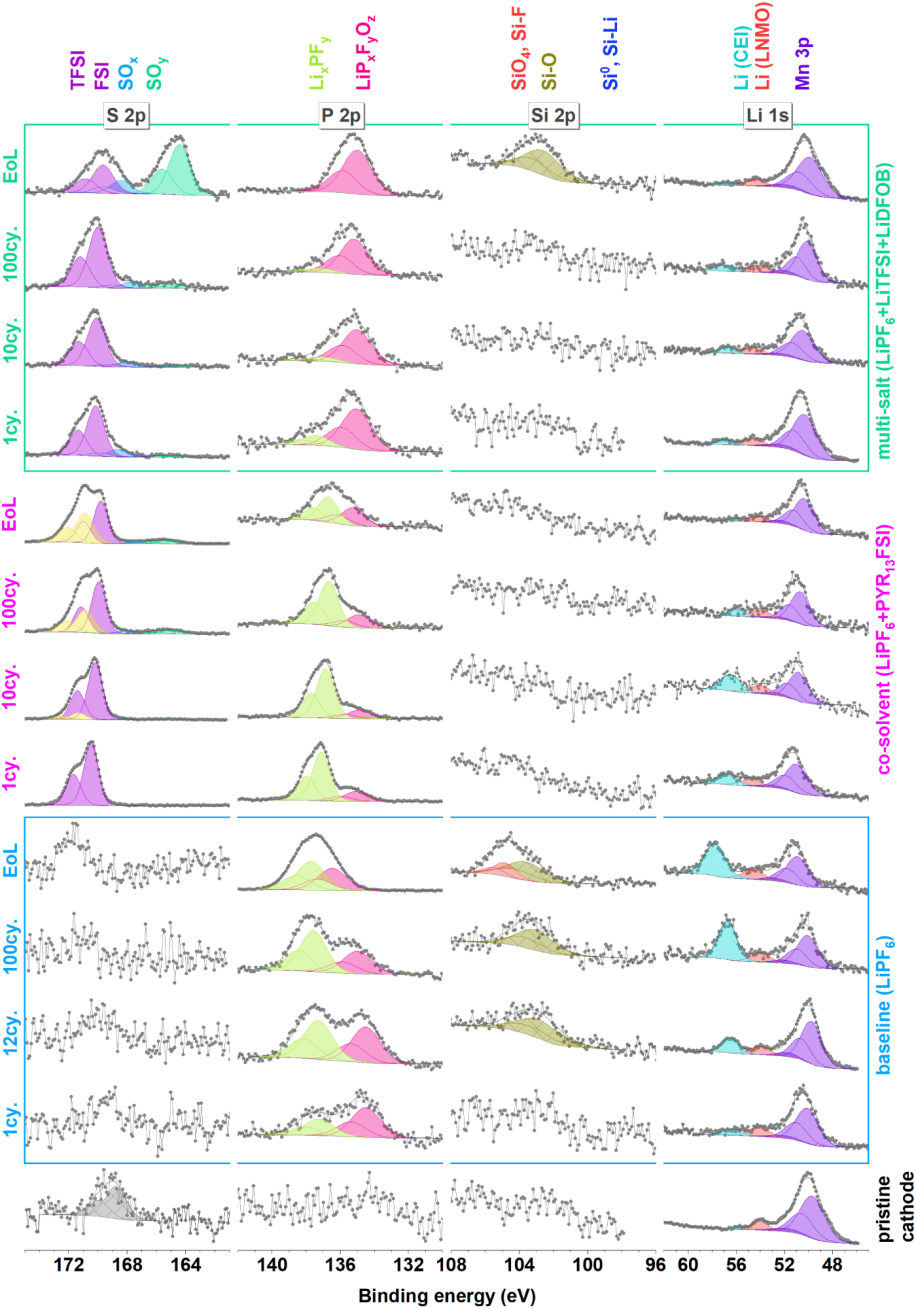
S 2p, P 2p, Si 2p, and Li 1s spectra of all positive electrodes.

All core-level spectra and their curve fits (details in the Experimental Details) with assigned peaks from the cathode are shown in Figures 2 and 3.

The observation of the conductive carbon (CB) C 1s peak (∼284.5 eV) in the pristine cathode as well as the cathodes in all stages of cycling shows that, in general, the CEIs formed for all electrolytes are relatively thin (Figure 2). The shift of the CB peak to higher binding energies compared to the pristine sample is unexpected but could be due to anion intercalation that can occur above 4.4 V vs Li^+^/Li. A similar shift has previously been observed in graphite samples by Kotronia et al.,^49^ and anions in the amorphous carbon black would likely have a similar effect on the C 1s peak position. However, the higher binding energy can also originate from a change in the relative electrochemical potential difference between the active material and the surface layer,^43^ as further discussed below. The relative ratios between C 1s peaks in the curve fit (assigned to carbon black and PVdF) stay stable at least until the 10th cycle for cathodes cycled by all electrolytes, indicating a CEI layer with a low amount of carbon-containing decomposition products. When compared to the pristine sample, the cathodes cycled in baseline and multi-salt electrolytes show slightly broader peaks, indicating a new surface chemical composition. On the 100th cycle and end-of-life cathodes, there is a clear buildup of a slightly thicker CEI, as seen from the decrease in this same ratio. In particular, CO_3_, CF_2_, and C–O peaks at C 1s spectra (290.8 and ∼286 eV, respectively) evolve for cathodes of the baseline and multi-salt electrolytes. The co-solvent electrolyte behaves differently so that it retains its carbon composition after the first cycle (indicated by almost identical C 1s spectra as in the initial cycles).

The CEI growth (observed by changes in LNMO and CB related XPS peaks) during the life span of the baseline and the multi-salt electrolyte is likely related to decomposition products from the organic solvent or FEC decomposition, providing C–F peaks or polyvinyl carbonate related peaks.^6^ CF_3_-related carbon compounds in the multi-salt sample can originate from the LiTFSI salt and are observed at all stages of the cycling around 293 eV in the C 1s spectra as well as in the F 1s spectra at ∼689–690 eV.

Multi-salt and co-solvent electrolytes show a LiF-related peak in F 1s spectra mainly at the EoL, suggesting that LiF only has a minor contribution to these formed CEIs. However, the high amount of LiF on baseline electrolyte’s CEI could originate from the crosstalk effect as discussed later in the paper.

The O 1s peak of LNMO (∼530 eV) is present at all stages of cycling. In the pristine electrode, the major LNMO component is located at 530 eV, and after the first cycle, this peak is shifted to higher binding energies (∼532 eV). With more cycling, this peak gradually shifts to lower binding energies of ∼1 eV in the 100th cycle. The shift of the peaks related to the (semiconductor) active cathode materials can be explained by the electrochemical potential difference between cycled and pristine electrodes as already explained by Lindgren et al.^50^ The similar shift in both CB (in C 1s) and LMNO (in O 1s) points in favor of this explanation. However, lately, it has also been more established to consider the involvement of oxygen as charge compensation, where an additional peak is fitted approximately ∼1 eV above the main metal oxide peak, of which the relative intensities vary with lithiation degree.^51^ To the best of our knowledge, this has not been studied for the LNMO-type materials, but in our case, this should give a minor effect, as the cathodes after cycling will have a similar lithium content. Assuming double-layer formation to be the cause of the shift in the metal oxide and CB, then the shift has to be very similar for the three electrolytes.

The dominating peaks in the range of 531–536 eV in the O 1s spectra of the cycled samples are assigned to carbonates, LiDFOB, PYR_13_FSI, and LiTFSI, and their decomposition products. At this range, the peak can be a superposition of several components that limit resolving individual oxygen contributions. Generally, it is expected to find the peaks of the O 1s and S 2p of the O=S=O group (from TFSI^–^) at a lower binding energy, than FSI^–^, due to the higher polarizing effect of the S–F bond relative to the S–CF_3_ bond of FSI^–^ and TFSI^–^, respectively.

**Figure 4:**
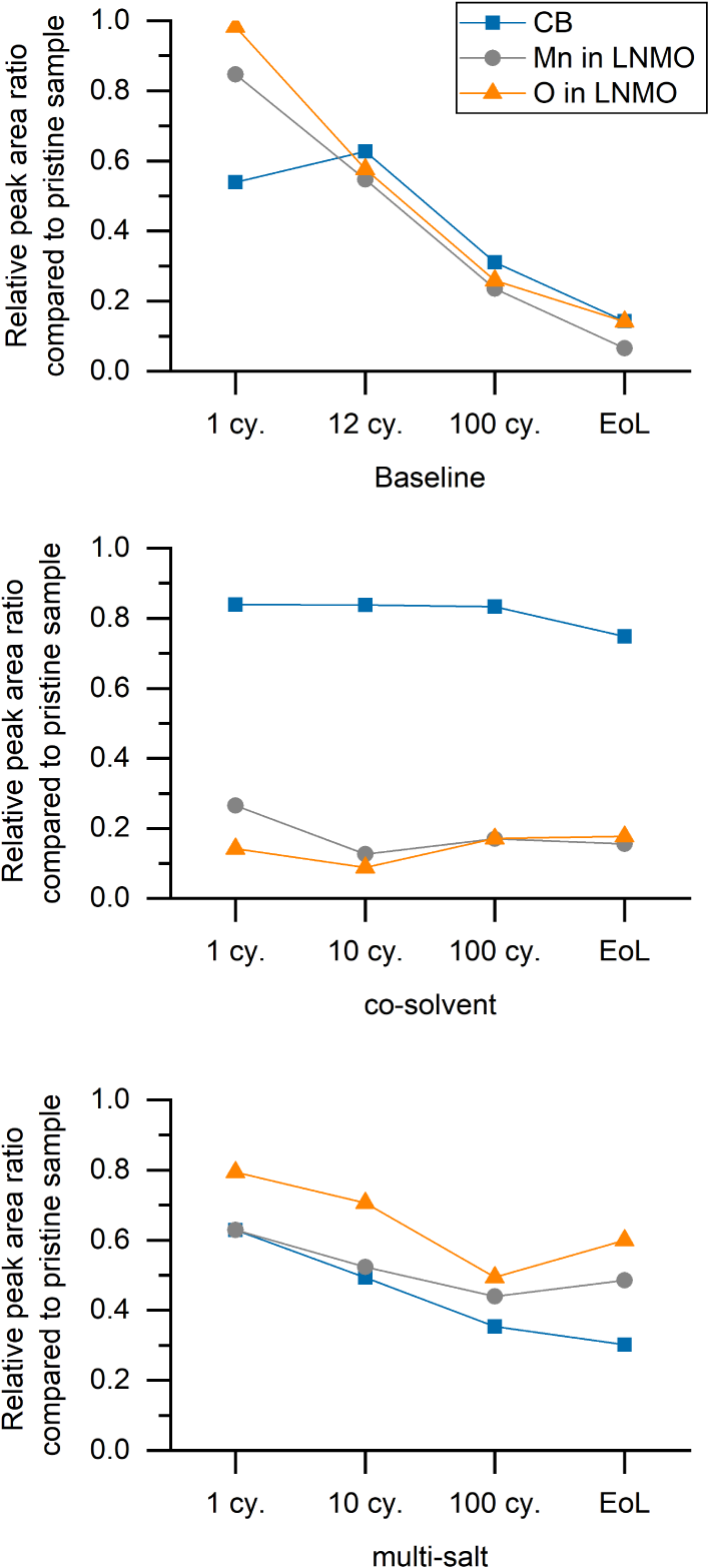
Relative intensity of CB (∼284.5 eV at C 1s), Mn 2p_3/2_, and O 1s of LNMO for each positive electrode at a specific cycle compared to their value in the pristine positive electrode.

Using the CB and LNMO signals from C 1s, Mn 2p, and O 1s (∼530 eV) spectra, it is possible to discuss the relative thickness of CEI layers and their evolution by increasing cycle number in more detail. Figure 4 shows the intensity of these chemical species normalized by their amount at the pristine positive electrode. It is apparent from Figure 4 that the baseline electrolyte initially shows relatively strong CB and LNMO signals that decrease with increasing cycling number and disappear by the EoL of the electrode. This indicates that the CEI builds up slowly but continuously over the lifetime of these cells. For the multi-salt electrolyte, a CEI of intermediate thickness forms on the LNMO after the first cycle. On subsequent cycles, the formed layer was observed to not build up as fast as seen in the case of the baseline electrolyte. Comparatively, for the co-solvent electrolyte, the LNMO-related peaks are already relatively small following the first cycle, and the peak intensity remains relatively consistent on subsequent cycles, suggesting a fairly thick CEI built by this electrolyte. However, while the consistent C 1s peak representing CB for the co-solvent electrolyte confirms a stable CEI up to the end-of-life, the relative intensity of this peak is notably high, suggesting a thin CEI, especially when compared to the low intensity of LNMO-related peaks at the same electrodes. At this point, we find no definite explanation for this phenomenon, but it can be speculated that the high intensity of the CB for co-solvent electrolyte may a consequence of unwashed PYR_13_FSI solvent or its decomposition products that contain C–N bonds, overlapping with the CB signal and subsequently shifted to higher binding energies (as discussed above). Another reason may be the reactivity of LNMO toward the co-solvent electrolyte, which leads to preferentially thick CEI on LNMO but simultaneously a thin CEI for the CB component, thus showing a CEI with varying thickness (uneven).

#### 3.2.2 Cross-Talk from the Si/G

It is valuable to observe and discuss the cross-talk that is apparent in these cells before dipping into the CEI composition. It is obvious that Si species are transferred from the Si/g electrode to the LNMO side in the Si 2p spectra shown in Figure 3. This is especially evident for the baseline electrolyte, where after 10 cycles, signs of silicon oxide or silicon oxyfluoride compounds are observed in the XPS-spectra from the LNMO surface. The Si 2p signal hereafter increases, and by the end-of-life, an additional Si 2p component is observed at higher binding energies. Also, using the multi-salt electrolyte, silicon compounds are observed at the LNMO surface at the end-of-life while the co-solvent electrolyte shows no indication of Si cross-talk.

Since it is obvious that Si species are transferred to the LNMO side, especially using the baseline electrolyte, it is interesting to elucidate what other species also have their origin in the Si/g electrode or its SEI. One such compound is likely LiF that normally does not form at the potentials of the LNMO electrode.^52−54^ In the case of the baseline electrolyte, the LiF peak increases up to the 100th cycle but disappears at the end-of-life, see F 1s spectra in Figure 2. Also, the multi-salt electrolyte shows an increase in LiF with cycling, although at much lower rates, while the co-solvent electrolyte indicates negligible relative amounts. The Li 1s peaks in the baseline electrolyte follow the same trend and increase on additional cycles. This would partially be explained by the increasing LiF content as well as the inclusion of intact LiPF_6_ molecules in the CEI. Notably, comparing the relative intensity of the Li 1s at around 56.5 eV attributed to Li in the CEI (Li–O, Li–F, Li–P, etc.) to Mn 2p content reveals some observations. The amount of Li on the cathode, seen from the relative intensity between Li 1s and Mn 2p, increases on cycling for the baseline electrolyte. This electrolyte also has a significantly higher amount of Li in its CEI compared to the other two electrolytes. A large part of this may stem from the fact that the CEI of the baseline electrolyte is thicker, thus reflecting the total amount of lithium ions that reside in the CEI. Part of this high Li content should be, however, explained by the LiF, but a larger fraction could also result from Li-carbonate compounds originally formed on the Si/g side that due to the cross-talk end up in the CEI. The multi-salt electrolyte produces a CEI with lower Li content, which appears more stable over time. For the co-solvent electrolyte, the amount of Li is generally low but experiences an increase in the 10th cycle. This increase in the Li peak cannot be ascribed to an increase in surface thickness as the thickness of the CEI remains relatively stable with an increasing cycle number for the co-solvent electrolyte. It is challenging to deduce if the Li intensity change is due to the dissolution of CEI species or because dissolved Mn species are moving further out of the CEI layers or due to a buildup of non-Li-containing CEI products. We will show in the next section that there is cross-talk of Mn species to the Si/g side, which means that Mn-containing species will have to pass through the CEI layers, and the spatial positioning of these species may affect the relative concentrations observed in the Li 1s spectra of cathodes.

Finally, emerging peaks that are typical for SEI layers (C–H, C–O, C=O containing compounds) can be found in the C 1s spectra for the multi-salt electrolyte and especially for the baseline electrolyte. This indicates that organic compounds are transferred between these electrolytes, while the co-solvent electrolyte exhibits much fewer of these organic species in the CEI. Based on the above-discussed extensive cross-talk from the anode to the cathode, maybe the electrochemical side reactions that occur on the LNMO cathode when cycling with the baseline and multi-salt electrolyte be very well comparable; however, cross-talk plays a crucial role in the continuous CEI layer buildup by these electrolytes.

#### 3.2.3 The CEI Composition

Due to cross-talk between the electrodes, it is challenging to deduce the origin of the decomposition products on each electrode. However, after the first cycle, cross-talk should be at a minimum level, and also the major CEI formation should have occurred. Studying the P 2p spectra (Figure 3) from the LiPF_6_ salt, which is used in all three electrolytes, reveals some differences. After the first cycle, it can be observed that the multi-salt electrolyte exhibits decomposition of the PF_6_^–^ anion on the cathode, as the major P 2p peak can be found at low binding energies (∼135 eV). The relative intensity from this peak continues to increase with increasing cycle number, indicating continuous salt decomposition and incorporation in the CEI. For the baseline electrolyte, relatively more of the intact salt molecules are present, and for the co-solvent electrolyte, only a minor peak of decomposed LiPF_6_ salt is observed. This again confirms HF trapping and cross-talk limiting property of co-solvent electrolyte formulation.

The LiTFSI salt stability in the multi-salt electrolyte is best represented by the S 2p peak, which shows only minor decomposition until the end-of-life. At the end-of-life, one major and one minor peak of reduced sulfur species suddenly appear. It is challenging to conclude whether this peak originates from decomposition at the LNMO electrode or if it is due to cross-talk from the Si/g electrode. However, the relative intensity of this peak is stronger at the LNMO side, which could indicate that it is formed there, and the formation of these decomposition products is accelerated toward the EoL, when the working potential of this electrode increases. The observation of the Al 2p peak is also suggested to be linked to this voltage increase at the EoL (see Figures 7 and 8). The LiTFSI and LiFSI salts are known to corrode the Al current collector at high voltages, and the corrosion products are then leached into the electrolyte and deposited on the LNMO surface.^19,20^ However, this unwanted side reaction is inhibited by using the co-solvent electrolyte formulation, which possibly has an optimal concentration of FSI^–^ to avoid Al etching. The S 2p peaks of PYR_13_FSI for the co-solvent electrolyte, however, seem to be more oxidized than reduced, since by increasing cycle number, an S 2p peak appears at higher binding energy relative to the first cycle; this can also be linked to the increased cathode’s working potential. Unfortunately, the role of LiDFOB in the multi-salt electrolyte could not be studied, since its characteristic peak (B 1s) overlaps with the higher intensity S 2s peaks.

The Li 1s peak at around 54.5 eV can be attributed to Li in the LNMO lattice structure. The normalization of this peak in different electrodes to their Mn content indicates that a lower amount of Li is observed on the surface of the LNMO material cycled by the co-solvent electrolyte. This fact together with the already discussed (uneven) thick CEI formation on LNMO active material cycled by the co-solvent electrolyte strengthens the plausibility of the suggested hypothesis on degradation of LNMO by the co-solvent electrolyte.

#### 3.2.4 The SEI Thickness

**Figure 5:**
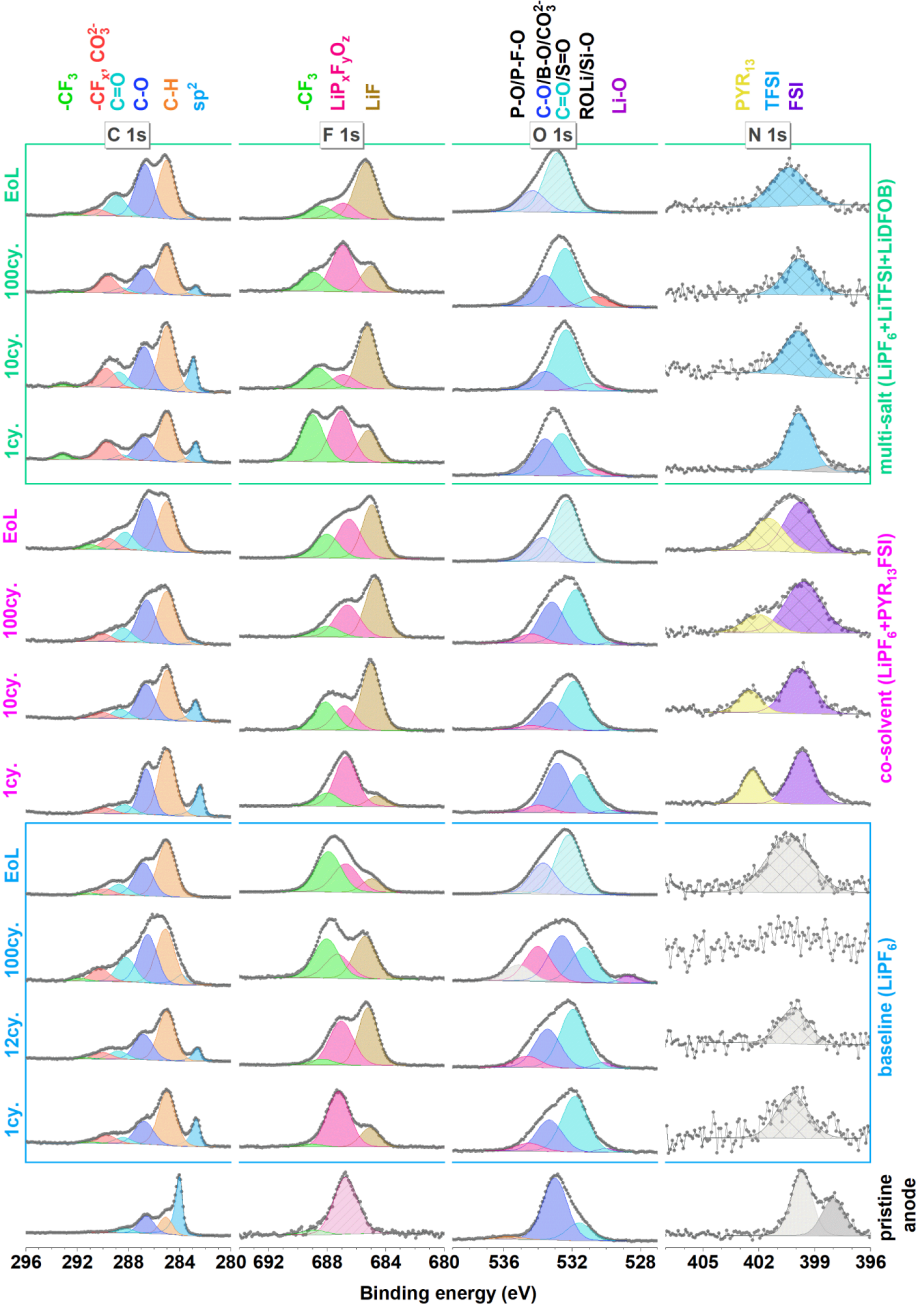
C 1s, F 1s, O 1s, and N 1s spectra of all negative electrodes.

For all the electrolytes, the graphite/CB peak of the Si/g electrodes shifts to lower binding energies after the first cycle due to the SEI formation^55^ (see Figure 5). The intensity of this peak is, in general, reduced with more cycles. While this peak can still clearly be observed for anodes cycled in the multi-salt electrolyte after the 100th cycle, it is barely visible for anodes cycled in the co-solvent electrolyte. Using this signal as an indicator of the SEI thickness, it can be argued that the SEI formed when using the baseline electrolyte grows faster (and becomes thicker) than the co-solvent electrolyte, which grows, in turn, faster than the multi-salt electrolyte.

**Figure 6:**
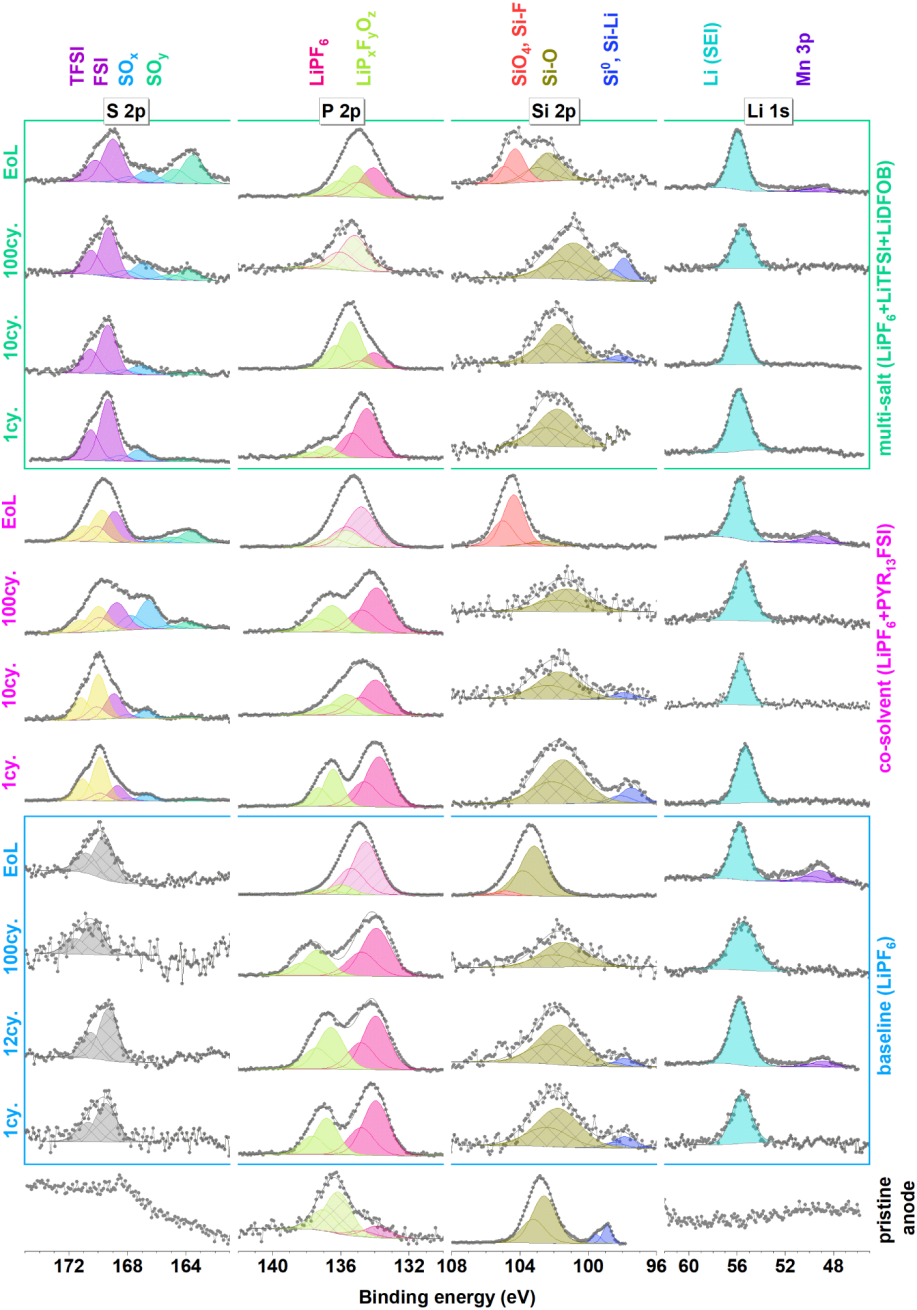
S 2p, P 2p, Si 2p, and Li 1s spectra of all negative electrodes.

The SEI formation also affects the signal from the Si in the anode, as shown in Figure 6, and the Si 2p intensity is becoming noisy already after the first cycle, making interpretations more challenging. Nonetheless, it was earlier shown that silicon oxide or silicon fluoride species are transferred to the LNMO electrode (see Si 2p spectra in Figure 3), and this implies that at some stage, they have to pass through the SEI layer. Thus, it makes sense that the observed Si 2p peak in the SEI is to some extent the material dissolved from the Si/g composite. This is especially true in the cases where the graphite peak intensity (indicating the level of active material contribution) is low. This can explain why the relative intensity of the metallic silicon to SiO_*x*_ continuously decreases in the case of the baseline and co-solvent electrolytes; simply because there are more and more SiO_*x*_/SiO_*x*_F_*y*_ species in the SEI.^56^ However, the opposite trend is observed for the multi-salt electrolyte and the relative signal from the metallic silicon actually increases up until the 100th cycle and suddenly disappears at the end-of-life. As mentioned for multi-salt electrolyte, until the 100th cycle, the decreasing graphite peak indicates that the SEI thickness increases; however, the increase in metallic Si 2p peak (which is also a signal of bulk active material) suggests that the SiO_*x*_ compounds on the surface are gradually reduced to metallic Si.^57^ This reduction to the metallic state can possibly show a slightly conductive SEI built by the multi-salt electrolyte. The following reaction can be suggested as responsible for the change: SiO*_x_* + Li^+^ + e^–^ → Si + LiO_*x*_. This reaction is, however, expected to leave Li_2_O in the SEI, nevertheless, a Li_2_O indication can only clearly be observed in O 1s (∼528 eV) spectra of the baseline electrolyte after 100 cycles as shown in Figure 5. The fact that it is not observed anywhere else is probably due to the reason that it can react further by participating in side reactions with the electrolyte, forming LiF, Li_2_CO_3_, and/or other SEI components^58^ or that the signal is very low in comparison to the other oxygen-containing compounds. Furthermore, a low intensity peak at around 530.5 eV in the O 1s spectra of the anodes cycled by the multi-salt electrolyte can be assigned to Li_2_O_2_ formation as a result of SiO_*x*_ reduction.

#### 3.2.5 The SEI Composition

The elemental composition of the SEI is, in descending order, dominated by C, O, Li, and F. Since these are the most frequent elements in the cells, it is perhaps not surprising that they build up between 85 and 95% of the SEI layer. Additionally, in the cases where the co-solvent and the multi-salt bring in further elements, only minor differences in the elemental ratios are observed. However, these minor differences may be crucial for the SEI functionality.

Since all electrolytes studied contain the FEC additive and the solvent in common, it is not surprising that the C 1s spectra for the different electrolyte systems show similarities. Nonetheless, it is possible to distinguish differences such as the relatively larger contribution of −CO_3_/CF_*x*_ compounds for anode cycled in the multi-salt electrolyte. It is also apparent from the high binding energy peak in the F 1s spectra that a unique compound is present in the SEI from the multi-salt electrolyte. The origin of this peak at F 1s spectra is either an unidentified compound or could possibly be attributed to the BF_2_ group on the LiDFOB additive.^59^ Also, the O 1s spectra of multi-salt electrolytes have a major component at ∼533 eV. This is possibly linked to the presence of LiDFOB salt or carbonates, which in turn supports that the O 1s spectra for this electrolyte are more dominated by salt molecules and their decomposition products and, thus, are comprised of relatively more inorganic compounds.^60^ This observation is supported by the work of Sun et al.,^61^ which utilizes the same salts as used in the multi-salt electrolyte in a Li/Li[Ni_0.59_Co_0.2_Mn_0.2_Al_0.01_]O_2_ cell setup. This work^61^ reported a multitude of inorganic compounds such as LiF, Li_2_CO_3_, Li_3_N, Li_2_S, and cross-linked O–B–O oligomeric and glass borates that were suggested to be beneficial for the passivating properties of the SEI. Despite the different cell chemistry in the mentioned study,^61^ the salts of the multi-salt electrolyte are shown to provide an inorganic-rich SEI, which decreases the decomposition of organic solvents.

In the O 1s spectra of the negative electrodes, a minor signal between 530 and 531 eV is observed until the 100th cycle. This is especially clear in anodes where the multi-salt and baseline electrolytes are used. In the case of the multi-salt electrolyte, this peak may be related to the formation of Li_2_O_2_ by the reduction of the SiO_*x*_ compounds, as discussed above. However, this peak may also be an indication of lithium silicates present in the SEI, as the higher intensity of this O 1s peak appears to be linked to the presence of a more intense peak from Li_*x*_SiO_*y*_ compounds observed in the Si 2p spectra for both multi-salt and baseline electrolytes.

The O 1s spectra of all of the end-of-life anodes are shifted slightly to higher binding energies and look very similar regardless of the electrolyte used. This is also true for other elements’ spectra such as Si, P, S, and C, which may bring us to the conclusion of the formation of similar SEI for EoL samples and this being responsible for the total capacity loss of these Si/g anodes.

Moreover, the intensive LiF formation on the anodes may refer to the fact that while the co-solvent and multi-salt electrolytes do not stop LiPF_6_ hydrolysis, they are relatively effective in trapping the produced HF and can hamper its damage to the electrodes while also better mitigating cross-talk.

Contrary to the LNMO electrodes cycled with the co-solvent electrolyte, the Si/g electrodes do not show an almost 1:1 ratio of PYR_13_ and FSI in the SEI (see N 1s spectra in Figures 2 and 5). Even after the first cycle, this ratio is changed to 1:2 due to the consumption or decomposition of the electrolyte. The decreasing ratio between the N 1s peak of PYR_13_ and the sum of the C 1s SEI peak areas (0.03, 0.02, and 0.02 for 1st, 10th, and 100th cycles), as compared to an increasing ratio for the FSI (0.05, 0.05, and 0.08 for 1st, 10th, and 100th cycles), indicates that it is primarily the PYR_13_ (peak at 402.5 eV) that decomposes into a compound with binding energy coinciding with that of FSI at 400 eV. Another interesting observation is the gradual lowering of the binding energy of the N 1s peak assigned to PYR_13_ with increasing cycle number. References^62−67^ indicate decomposition reactions for PYR_13_ that give rise to an organic SEI layer. This observation of the gradual decomposition of PYR_13_ may also be linked to the high amount of CB peak seen for cathodes cycled in the co-solvent electrolyte, in a way that the CB peak has overlapping signals originating from PYR_13_ decomposition products at the anodes. Due to the cross-talk between electrodes, the PYR_13_ decomposition products could possibly migrate to the cathode and contribute to the curve-fitted CB peak as C–H or C–N-containing compounds.

The sulfur of the FSI anion in the co-solvent electrolyte behaves differently than the TFSI anion from the multi-salt electrolyte, similar to what is described in the cathode. However, on the anodes cycled in the co-solvent electrolyte, the highest binding energy peak does not gain in intensity with more cycles, meaning that FSI decomposes differently than it was observed to do so on the cathodes. Although the S 2p peak of LiTFSI is accompanied by excessive decomposition, as can be seen by emerging S 2p peaks at around 167 and 164 eV, there are no major changes to the N 1s peak of LiTFSI except toward the end-of-life, where a minor shift and broadening of the peaks are observed. This result suggests different fragmentation or bond breaking of LiTFSI in comparison to PYR_13_FSI on both electrodes, which may influence the SEI and CEI properties.

Finally, a general observation for all of the electrolytes indicates some Si species at a high binding energy (∼104 eV) for end-of-life anodes. These species should be located in the SEI due to the lack of bulk graphite peak observation for end-of-life anodes. This high binding energy suggests that more electronegative elements are now surrounding the Si atoms, and it can be assumed that silicon fluoride compounds have been formed by the end-of-life.^20,22,27^ These spectra thereby show that a considerable change in the Si chemistry occurs at the end-of-life, and more investigation is needed to clarify the origin of this observation.

#### 3.2.6 Cross-Talk from the LNMO

**Figure 7:**
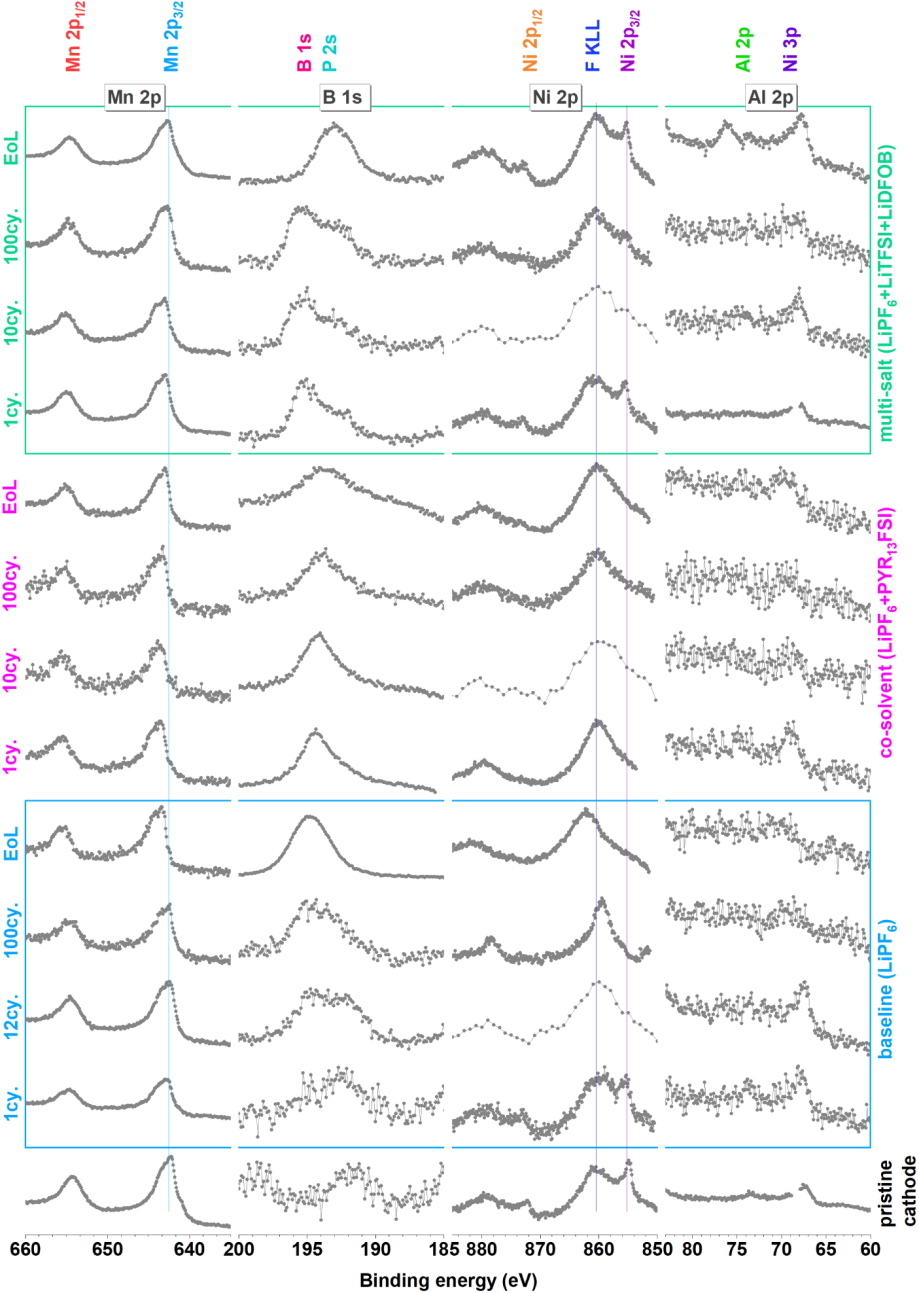
Mn 2p, B 1s, Ni 2p, and Al 2p spectra of all positive electrodes.

**Figure 8:**
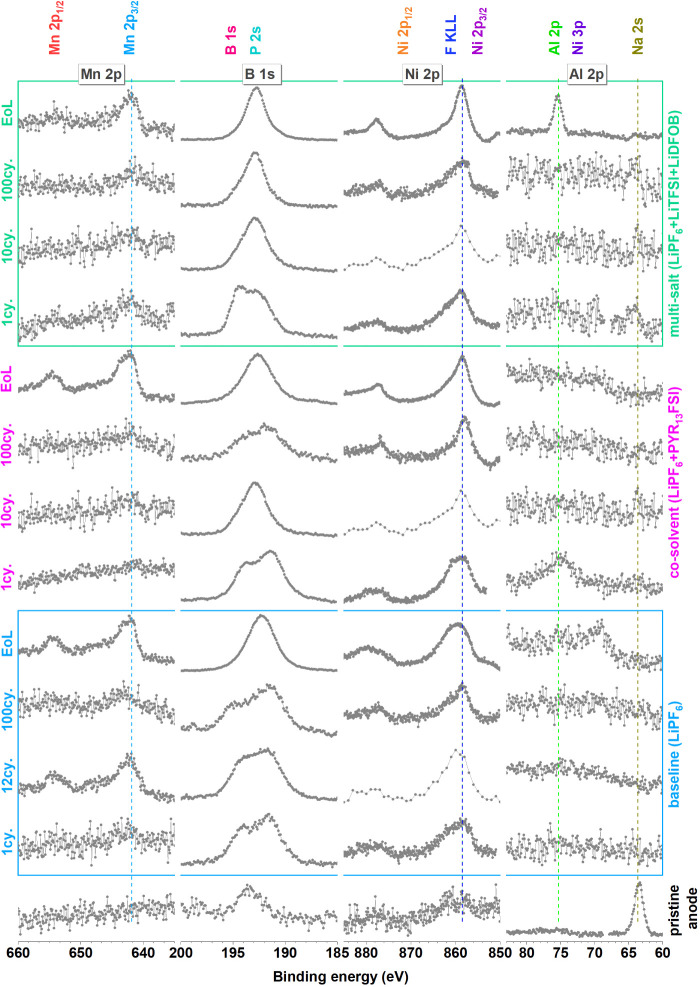
Mn 2p, B 1s, Ni 2p, and Al 2p spectra of all negative electrodes.

In early cycles, especially for the baseline electrolyte, a considerable signal from Mn is observed in the SEI (Figures 7 and 8). By the end-of-life, all electrolyte systems reveal a strong Mn peak on anodes, which confirms that a considerable amount of Mn dissolution from the LNMO occurs for all electrolytes investigated. This drastic increase of Mn on the anodes toward the end-of-life is possibly related to the increased working potential for the LNMO electrode, rather than being just a consequence of the time, the LNMO is kept in the electrolyte.

## 4 Summary and Conclusions

**Figure 9:**
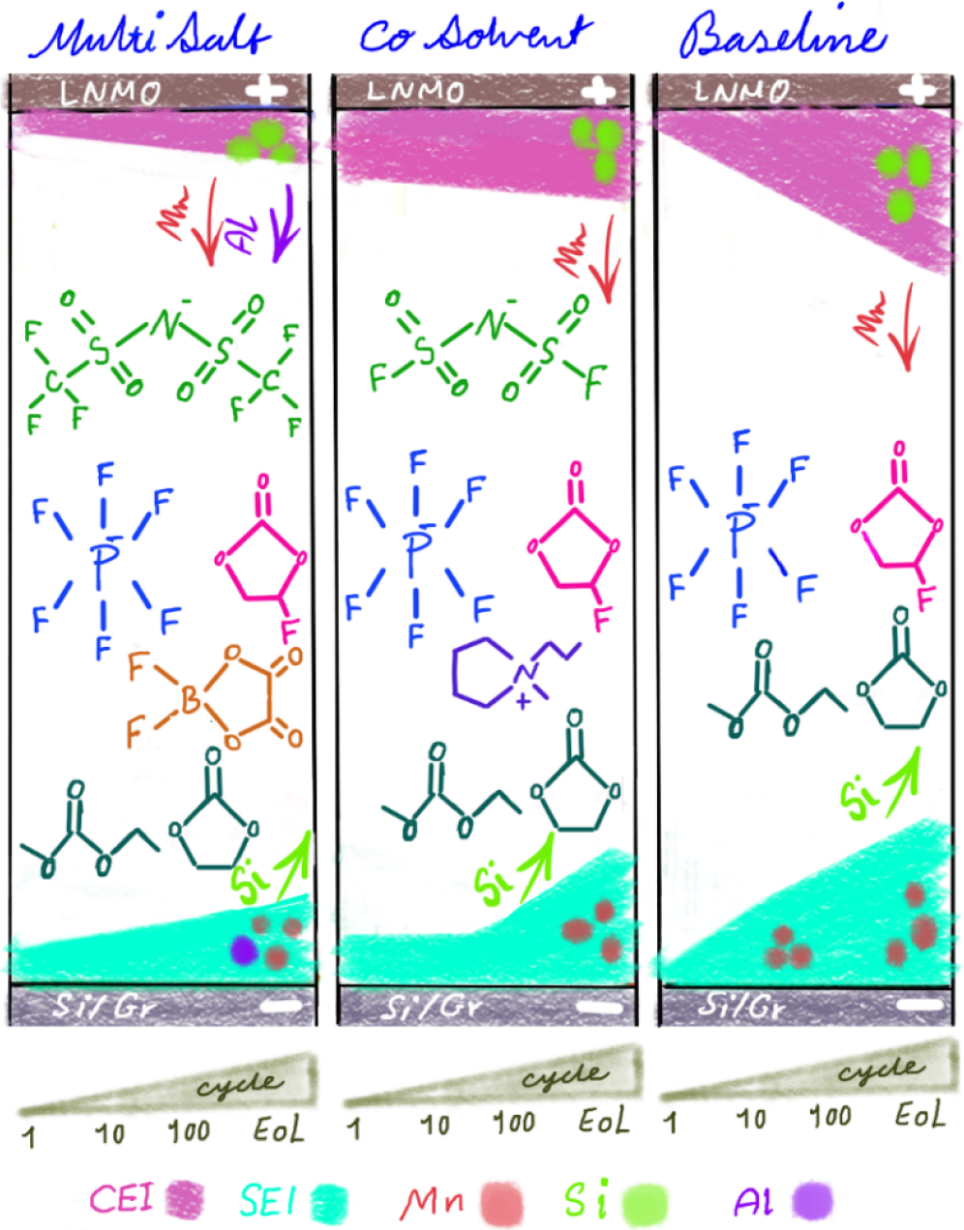
Schematic summary representing SEI and CEI thickness and detected major degradation mechanism.

In this study, we have used XPS to trace side reactions and surface layer formation in different electrolytes, as summarized in Figure 9. More specifically, we have compared the effect of using salt additives (multiple salts) to the effect of using ionic liquid solvent additives and compared those to a standard electrolyte to investigate side reactions in high-voltage LNMO vs Si/g full-cell lithium-ion batteries. Several well-known detrimental side reactions are observed and elucidated. The cycling results reveal that the reproducibility of cycling performance is affected to a large degree by the ratio of electrode capacities (cell balancing) and the side reactions of the active electrode materials.

The thinnest SEI and CEI are observed using the multi-salt electrolyte, and its surface layers increase in thickness at a slower rate compared to the other electrolytes. This contrasts to the observations for the baseline electrolyte, which, while initially yielding a thin SEI and CEI, grows thicker and at a quicker rate upon continued cycling.

The LNMO positive electrode reveals the dissolution of manganese, which is transferred to the SEI on the negative electrode. This effect is most pronounced using the multi-salt and the baseline electrolyte but is less observable with the ionic liquid co-solvent electrolyte. Additionally, aluminum from the LNMO current collector is found in both the CEI and SEI when using the multi-salt electrolyte, while it was absent when using the other electrolytes. The Si/g electrode exhibits dissolution of silicon oxide species as they are found in both the SEI and the CEI especially for the baseline electrolyte but also at the end-of-life using the multi-salt electrolyte, and this side effect is prevented in the co-solvent electrolyte.

The CEI layer buildup is partly facilitated through the decomposition of electrolyte at the cathode itself but is also influenced by significant cross-talk from the Si/g electrode. The thickness of the CEI layer could thus be considered as a sum of the mechanisms ongoing in the cell. Nonetheless, in the cases where the CEI buildup is minimal, it should be fair to say that the cross-talk is relatively small, like in the case of the multi-salt and the co-solvent electrolyte. This emphasizes the need for tailored electrolytes with multiple components to satisfy the stabilization needs of Si/g and the high voltage cathodes to make it work.

The same argument seems to be less critical for the Si/g electrode as the decomposition products from the LNMO side are significantly fewer; however, the effects of Mn dissolution are obviously a factor that requires consideration. The SEI thickness observed in these measurements is then of course influenced by the amount of SEI dissolution into the electrolyte, and again this dissolution is evidently more critical in the case of the baseline electrolyte. Even though the dissolution is high, the SEI coverage in this electrolyte still diminishes the bulk graphite signal, indicating that this SEI is thickest. In this respect, it may be argued that the multi-salt and co-solvent electrolytes both contribute to a thinner and more flexible SEI, which can more effectively accommodate the volume change of Si/g anode and high voltages of the LNMO cathode. Moreover, the co-solvent electrolyte seems to suppress some of the cross-talk such as migration of Si, LiF, and organic compounds to cathodes.
